# Obfuscated Malware Detection and Classification in Network Traffic Leveraging Hybrid Large Language Models and Synthetic Data

**DOI:** 10.3390/s25010202

**Published:** 2025-01-01

**Authors:** Mehwish Naseer, Farhan Ullah, Samia Ijaz, Hamad Naeem, Amjad Alsirhani, Ghadah Naif Alwakid, Abdullah Alomari

**Affiliations:** 1 Computer and Software Engineering Department, College of Electrical and Mechanical Engineering, National University of Sciences and Technology (NUST), Islamabad 44080, Pakistan; mehwish.naseer@ceme.nust.edu.pk; 2 Cybersecurity Center, Prince Mohammad Bin Fahd University, 617, Al Jawharah, Khobar, Dhahran 34754, Saudi Arabia; farhankhan.cs@yahoo.com; 3 Computer Science Department, HITEC University, Taxila 47080, Pakistan; samia.ijaz@hitecuni.edu.pk; 4 Department of Computer Science, College of Computer Sciences and Information Technology (CCSIT), King Faisal University, P.O. Box 400, Al-Ahsa 31982, Saudi Arabia; haazaam@kfu.edu.sa; 5 Department of Computer Science, College of Computer and Information Sciences, Jouf University, Al Jouf 72388, Saudi Arabia; 6 Department of Computer Science, College of Computer and Information Sciences, Jouf University, Sakaka 72341, Saudi Arabia; gnalwakid@ju.edu.sa; 7 Department of Computer Science, Al-Baha University, Al-Baha 65779, Saudi Arabia; alomari@bu.edu.sa

**Keywords:** smart sensing, cybersecurity, large language models, malware classification, generative AI, transfer learning

## Abstract

Android malware detection remains a critical issue for mobile security. Cybercriminals target Android since it is the most popular smartphone operating system (OS). Malware detection, analysis, and classification have become diverse research areas. This paper presents a smart sensing model based on large language models (LLMs) for developing and classifying network traffic-based Android malware. The network traffic that constantly connects Android apps may contain harmful components that may damage these apps. However, one of the main challenges in developing smart sensing systems for malware analysis is the scarcity of traffic data due to privacy concerns. To overcome this, a two-step smart sensing model Syn-detect is proposed. The first step involves generating synthetic TCP malware traffic data with malicious content using GPT-2. These data are then preprocessed and used in the second step, which focuses on malware classification. This phase leverages a fine-tuned LLM, Bidirectional Encoder Representations from Transformers (BERT), with classification layers. BERT is responsible for tokenization, generating word embeddings, and classifying malware. The Syn-detect model was tested on two Android malware datasets: CIC-AndMal2017 and CIC-AAGM2017. The model achieved an accuracy of 99.8% on CIC-AndMal2017 and 99.3% on CIC-AAGM2017. The Matthew’s Correlation Coefficient (MCC) values for the predictions were 99% for CIC-AndMal2017 and 98% for CIC-AAGM2017. These results demonstrate the strong performance of the Syn-detect smart sensing model. Compared to the latest research in Android malware classification, the model outperformed other approaches, delivering promising results.

## 1. Introduction

Technology is progressing and becoming an essential part of everyday life. Cybersecurity threats to personal digital spaces are becoming increasingly critical and concerning, as digital systems face new malware every day [1]. Traditional malware detection and classification systems are mostly signature-based and may not identify the daily evolving patterns. Therefore, machine learning and AI-based models appear as new means to address the issue [2,3]. Malware is classified into different groups such as adware, ransomware, trojans, and SMS malware. The classification of malware is crucial as it shows the kind of harm each malware can cause to the system and how malware should be responded to by the system depending on the type of malicious attack [4]. Malware attacks may lead to mild to catastrophic losses to the systems [5]. Among the hand-held gadgets, Android is one of the most used operating systems in mobile phones [6]. Android-based apps are installed on mobile phones for several purposes such as online payment, medical record-keeping, gaming, and educational purposes [7]. As Android apps are more commonly used, they are more prone to malicious attacks [8].

### 1.1. AI in Malware Detection and Classification Paradigm

Due to the nascent patterns of malware, AI and machine learning proved themselves as successful methods for malware detection and classification. Malware classification is the subject of machine learning, and classifiers such as the Naïve Bayes Classifier, Logistic Regression, and K-Nearest Neighbors have been successfully deployed for the job [9]. Algorithms like Convolutional Neural Networks, Random Forests, Hidden Markov Models, and Support Vector Machines have been used in approaches to malware detection and classification [10]. However, as the models for malware detection are advancing, the attackers are becoming alert as well and machine learning algorithms are showing deteriorating performance. This is taking the domain of AI-based malware detection towards explainable AI and making it more challenging [11,12].

Similarly, natural language processing methods (NLP) are also taking their way into the threat detection paradigm [13]. NLP methods are being used for visualization of the internal structure of the malware pattern [14]. NLP extracts the keyword from the textual representation and grasps the semantics of the text. So, while finding the traces of malicious content in the network traffic, this approach is hope and interest of the researchers in the domain of malware detection [15]. NLP algorithms such as Bag of Words (BOW), Term Frequency-Inverse Document Frequency (TF-IDF) matrices, Word2vec, and FastText are promising in determining the patterns of malware data. Determining the sequential information is of prime concern in malware detection models; so, Word2vec and FastText are competitive approaches in the development of malware detection models [16,17]. NLP further expands the services with its larger deep learning models for textual data processing and classification tasks with transformer models such as BERT, RoBERTa [18], and DeBERTa [19]. Transformer models possess the capability to process large and complex data. So, these models are becoming popular for the provision of complex solutions such as malware detection datasets [20].

### 1.2. Challenges with Malware Detection and Classification

While collecting Android malware data, the scarcity of the different classes in the collected data is a challenge [21]. High-dimensional malware data and imbalanced data impact the performance of machine learning and deep learning models [22]. The dynamic and evolving nature of the malware is another key challenge for machine learning and deep learning models while being applied to malware detection systems [23]. The dataset plays a key role in the success of deep learning models. The availability of large datasets is prime to the success of NLP. In actual scenarios, imbalanced samples are faced by malware detection systems [24]. It is significant to understand the working of the models in such challenging scenarios [25]. One of the foremost pathways to harm the Android app is network connectivity. Since Android apps mostly work over the network, malicious content may travel to the apps through network traffic. So, malware detection and classification researchers are always concerned about looking up network traffic to realize the traces of malware. So, the approaches that use network traffic data for malware detection and classification discover the malware patterns for accurate classification practices [26]. A robust and intellectual malware detection system with high precision and recall for multiple malware types is a need of today’s digital society, where most daily life tasks are performed through screen touches on Android apps and malware patterns are evolving exponentially [27].

### 1.3. Contributions of the Current Study

Considering the impact of TCP flow in the network traffic for mining the traces of malware, we are focused toward developing an enhanced smart sensing malware detection and classification model through transformer-based NLP. The main contributions of the paper are as follows:

1. A custom dataset is prepared by extracting the TCP data flow from network traffic and unpacking packet data from a benchmark dataset. The features are then analyzed and transformed into a labeled dataset for the transformer model.
2. TCP synthetic data are created by generating contextual word embeddings for sentence augmentation at level 2. A transformer-based GPT-2 model is developed to address the challenge of scarcity in malware data.
3. An adapted BERT model is developed by incorporating classification layers for malware detection and classification. The features are extracted as word embeddings using a BERT Tokenizer.

A comprehensive literature review is conducted to evaluate the uses of transformers and LLMs in malware detection and classification. The literature review explains how LLMs are used to detect malware. Further, the challenges connected with the models are considered while defining research gaps. The research experiments are carried out using the Google Collaboratory Python notebook. The structure of the paper is as follows: Section 2 presents the related work. Section 3 explains the Syn-detect models’ architecture for malware classification. Section 4 provides an insight into the experiment and results. Section 5 presents the comparison of the results of the model, and Section 6 describes the conclusions of the research and future work. Declarations, contributions, and references are mentioned after that.

## 2. Related Work

LLM is a promising approach in the malware detection domain. Previous studies have utilized multiple models of LLM to achieve the goals due to their capability of contextual understanding of the textual datasets. Different websites may contain phishing content, and experiments with GPT-4 to detect phishing elements on these sites have yielded substantial results [28].

### 2.1. Transformers/LLMs in Malware Detection

Similarly, Android apps use LLM models to detect malware. Ref. [29] proposed a supervised learning model for the detection of malware in Android apps called Adroid-COCO. Program dependency graphs were developed from the byte and native code and used for Android malware detection on a large data sample; the approach performed well, with significant results of 99.8% accuracy. The MalBERT model was proposed by [30], which uses the transformer architecture of BERT for the detection of malware. The model performs static code analysis of the Android apps to identify the malicious content in the code. This model depicts the efficient utilization of the transformer architectures for malware detection by showing 97.6% accuracy for binary classification and 91.02% accuracy for multiclass classification. MalBERT v2 was presented as an extension of MalBERT by [31]. This model works on the source code of the apps and extracts the files with the most relevant information. The data are tokenized and classified using BERT to classify the data as Malware and Goodware. This model verifies the excellent performance of the BERT architecture by achieving the weighted F1-score of 82–99% on different public datasets. Another method for identifying harmful information is to extract text from Portable Executable (PE) files. Ref. [32] used this textual information to create two labeled datasets of benign and malware data and unlabeled test data. They used Stacked-BiLSTM (stacked bidirectional long short-term memory), DistilBERT, and DSLM (Domain Specific Language Model) GPT-2 (generative pre-trained transformer) for the analysis of the generated datasets to identify malware. The experiments on these models based on transformers achieved 76.2–98.3% F1-scores. The power of transformers for the detection of malware was further explored by [33]. They developed API call graphs generated from the Android API call graphs. These graphs are developed by determining the relationships and dependencies between different API calls. This resulted in categorizing the API call as malware or benign by showing promising results with higher accuracy.

### 2.2. Dataset Preparation for Malware Detection

Transformer and NLP model performance depends on threat detection dataset parameters. Malware is classified according to the behaviors it exhibits after entering the system. The datasets available for research are heavily skewed, and not all varieties of malware are equally distributed. This type of data leads to misclassification because the model struggles to discover trends or malware families when the sample size is small. Data augmentation is recommended to balance datasets. Ref. [34] experimented with extracting the features from the Application Program Interface (API) to analyze the families of malware according to their behavior. In the experiment, they augmented the data and used that for model creation for classification. The augmented dataset depicted the improved results of the classification with an F1-score of 91% and accuracy of 99%, as compared to the utilization of only synthetic data, which resulted in a 0.65 F1-score and 0.63% accuracy. For the same data balancing issue, [35] presented the BalBERT model. This approach addresses the use of transformer architecture for data balancing by incorporating more layers into BERT. The BalBERT model demonstrates beneficial results by having a higher average recall and F1-score than the baseline BERT model. This demonstrates the need for data balancing for classification models to achieve optimal performance. Ref. [36] also used BERT by oversampling the data of the minority classes and undersampling the majority classes. This resampling strategy for data balancing proved to be an excellent way to build word embeddings from BERT for efficient classification. Cohen’s kappa, accuracy, ROC-AUC curve, and MCC, as evaluation metrics with k-fold validation, are used to demonstrate improved performance. Ref. [37] used network traffic data patterns for the identification of malware in PCAP files. They employed GANs to generate synthetic samples of traffic data, which produced similar results. This introduced CNN GAN in cybersecurity, which uses network traffic data encoding into image-based matrix representations to assess numerous vulnerabilities. The PAC-GPT model is another innovative approach to the generation of synthetic data from packet data using GPT-3. The performance of this packet generator is examined using several assessments and reveals that transformers are an appropriate solution for synthetic packet creation with minimal fine-tuning performed [38].

The literature study emphasizes the need to use various strategies to generate synthetic samples to increase classification accuracy. The significance of the generation of synthetic traffic data samples for malware identification, however, is not assessed by any method. The examination of the literature also suggests that BERT is a successful transformer architecture for malware detection. The Syn-detect approach attempts to identify network malware using synthetic packet data. The model generates packet data using GPT-2, and the synthetic samples are supplemented with raw samples. These new data are placed into the BERT tokenizer to generate word embeddings, which are then fed into an updated BERT model with extra layers that classify malware into different families.

## 3. Methodology

**Figure 1:**
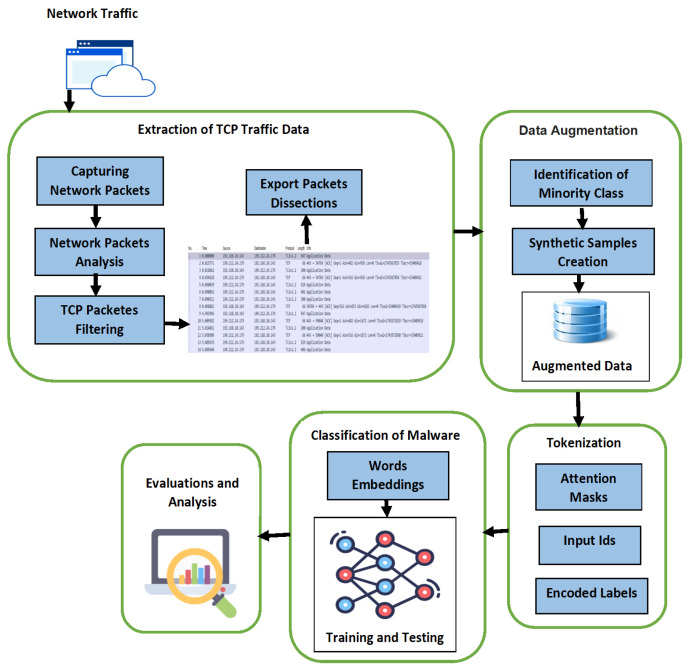
Syn-detect model of malware classification.

The overall framework of the Syn-detect model is represented in Figure 1. Figure 1 illustrates the process of malware classification through the LLM-based hybrid model involving the extraction of TCP protocol segments of network traffic data of Android Apps from PCAP files. Then, TCP data are fed into the synthetic data generator to generate the synthetic samples of the minority class and balance the data. These samples are augmented with the raw data to generate a new dataset, which is employed as the input to the tokenizer after preprocessing. Tokenizer transforms the textual data into tokens. This representation is an acceptable format for the transformer. The adapted BERT model is fine-tuned to classify the data into malware families. Evaluations of the results revealed how well the Syn-detect model works.

### 3.1. Extraction of TCP Traffic Data

Network traffic data are collected in the form of PCAP files. Network traffic is assembled in the form of 5-tuple data having a source IP, destination IP, source port, destination port, and protocol. Table 1 illustrates the significance of each attribute in the traffic data.

We used Wireshark to read the PCAP data. The packet data are extracted through the export packet dissections operation in Wireshark [39]. The traffic flows from the sender to the receiver are determined by all of the attributes listed in Table 1. Among all these criteria, we choose the protocol to select the data. Communication protocols include UPD, TCP, and HTTP. Protocols define the mechanism of communication and data transfer between sender and receiver. TCP is a vital protocol for network services and applications [40]. TCP allows in-depth analysis of the packet sequences, which helps in examining the malicious content during communication. So, for the data collection, we extracted the TCP data packets from the network traffic. Algorithm 1 represents the mechanism for the extraction of TCP packet data from the network traffic.
**Algorithm 1** Extraction of TCP Traffic Data**Input:** Network Traffic Data Pc**Output:** TCP Traffic Data TCP_Pc**Initialization:**1:    TCP_Pc = {}2: **For** each packet Pc_i in Pc do:3:    Protocol = ExtractProtocol(Pc_i)4:    **If** Protocol == ‘TCP’ **then:**5:         Add Pc_i to TCP_Pc6: **Return** TCP_Pc

### 3.2. Synthetic Sample Generator and Data Augmentation

The retrieved TCP packet dataset is evaluated to identify a candidate class for synthetic data generation. The classifier misclassifies samples that are underrepresented in the dataset. Lack of learning examples from these classes and reduced precision and recall reduce classifier efficiency. Classes with fewer than the average number of samples are classified as minority and sentence augmentation is conducted on samples from that class. Sentence augmentation level 2 results in the generation of two similar sentences [41]. GPT-2 from NLPAug library (1.1.11) of hugging face is used to generate the synthetic samples keeping the level of generation to 2 [42]. Once the minority classes have been identified and new samples obtained, the new data are combined with existing information to produce the final dataset. Algorithm 2 depicts the mechanism of the synthetic sample generator and the augmentation process for generating the custom dataset.
**Algorithm 2** Synthetic Sample Generator and Data Augmentation**Input:** TCP Traffic Data TCP_Pc**Output:** Augmented Traffic Data Aug_Pc**Initialization:**1: Aug_Pc = {}2: **For** each Class C_Pc in TCP_Pc do:3:    Packet_Pc = Count C_Pc4:    **If** Packet_Pc < ‘Threshold’5:    AugPacket_Pc = Generate2Samples for Packet_Pc **then:**6:     Aug_Pc = AugPacket_Pc U TCP_Pc7: **Return** Aug_Pc

The quality of the synthetic data generated by GPT-2 was thoroughly validated by calculating the Kullback–Leibler (KL) divergence between the synthetic data and actual traffic data [43]. KL divergence provides a quantitative measure of how the probability distribution of the synthetic traffic data diverges from that of the original traffic data, which helps us to evaluate the realism and diversity of the generated samples. This analysis helps to prove the effectiveness of the synthetic traffic data in approximating real-world traffic patterns. To compute the KL divergence of traffic data, we vectorized the traffic data using TF-IDF. Once the two vectors are obtained as original traffic data and synthetic traffic data, the KL divergence between the original text data distribution *P* and the synthetic text data distribution *Q*, based on their TF-IDF values, is given by Equation (1):

(1) $$D_{KL}\left(P\|Q\right)=\sum_{i}P\left(t_{i}\right)\log\frac{P(t_{i})}{Q(t_{i})}$$

where we have the following:

- $P(t_{i})$ is the TF-IDF value of term $t_{i}$ in the original text data;
- $Q(t_{i})$ is the TF-IDF value of term $t_{i}$ in the synthetic text data;
- The sum is taken over all terms $t_{i}$ in the vocabulary.

### 3.3. Ethical Considerations with Synthetic Data

The use of synthetic data provides an opportunity to accelerate research and model training. However, the responsible use of synthetic data requires attention regarding expected risks and challenges. Since synthetic data mimic the original data, it is vital to keep ethical issues in consideration. A report by [44] guides how practitioners and innovators can responsibly use synthetic data. The synthetic malware traffic samples developed in this study are aimed at improving the performance of models used in cybersecurity research. The synthetic malware dataset will not be publicly released. It will only be used internally for research purposes or shared with the trusted researchers who are involved in the project. We aim to prevent any potential harm that could arise from the creation of synthetic data.

### 3.4. Features Analysis

**Figure 2:**
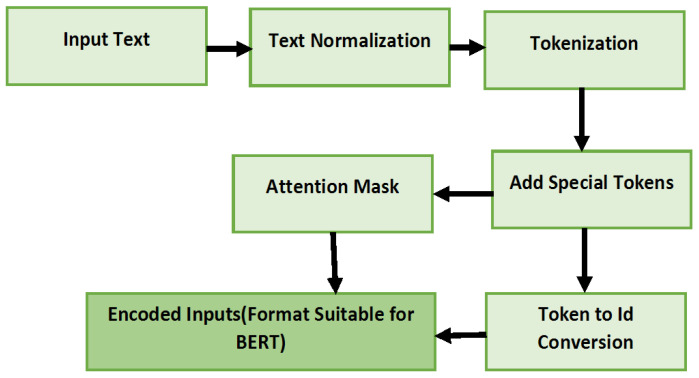
Features analysis process.

The augmented data generated by the previous step undergo preprocessing with the NLTK library to remove the spaces and NULL values in the dataset [45]. The preprocessed data are further fed into the Tokenizer. Tokenization converts the textual data into a format that is understandable by the LLM model. BERT Tokenizer from Transformers library (version 4.42.4) is used in this model to perform the tokenization [46]. Tokenizer converts the data into smaller tokens. BERT Tokenizer generates tokens based on the word piece policy. With this process, each sentence splits into a word. Special tokens [CLS], [SEP], and [Pad] are added with Tokens. These Tokens help in the efficient handling of the text by maintaining a consistent representation of the text for the model to perform the classification. Attention Masks are used by BERT tokenizer to mask the originally generated token from the padded one. Token IDs for each token are generated by the tokenizer. Ultimately, BERT Tokenizer generates the Attention Mask and Token IDs as outputs. This is the Input for the BERT model along with the encoded labels for the classification. Figure 2 illustrates the process of Tokenization by the BERT tokenizer used in the Syn-detect model for tokenization.

### 3.5. Classification and Evaluation

BERT is a pre-trained model for textual data bidirectional representations [47,48]. Additional layers can be added to BERT to fine-tune it to perform the specific task of NLP such as classification. BERT is a state-of-the-art model and proved its computational and empirical power in research on NLP. So, BERT is fine-tuned through hyperparameter adjustments and the inclusion of new layers to perform the malware classification in the Syn-detect model. The BERT unit of the model’s structure is explained below [49]:

1. The 1st layer of the model is the input layer. This layer receives the input in the form of Attention Mask and Input ID. The required input is generated by the Tokenizer from the textual data. Tensorflow is used for model representation in Python so that input in the form of tensors is accepted by the model [50]. The shape of the input tensor is precise to 256 tokens; this resulted in the padding of the shorter token and truncation of larger tokens to keep the consistency of input.
2. The input is fed to the pre-trained BERT model—a BERT-based uncased model from the Transformers library (4.42.4). Data are processed by BERT, resulting in two major outputs. Activation layer and Pooled Output layer. For the classification through BERT, a sequence to be used for classification by the Pooled Output Layer is further input to the new layers designed for classifications.
3. An intermediate Dense layer is included, which is a fully connected dense layer, using the Rectified Linear Unit (ReLU) as an activation function to enhance the learning capability of the model. This layer is designed for 512 units, so it helps in dimensionality reductions of the features generated by the BERT pooled output layer, keeping the imperative information required for classification integral.
4. Following that, another dense layer, the output layer, is added to perform the classification and generate the probability distribution of the classes. The SoftMax activation function is used to calculate the probabilities of the classes using classification.

**Figure 3:**
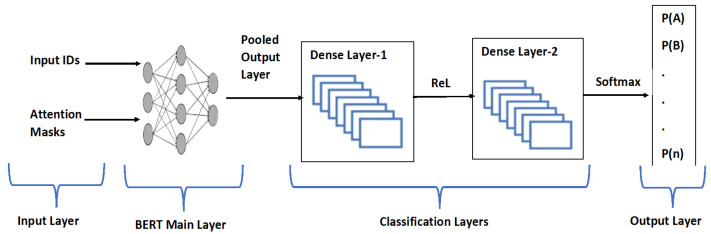
Adapted BERT model in Syn-detect.

The designed model utilizes the powerful embeddings generated by BERT and additional layers to perform the classification over multiclass data. The adapted BERT model for the proposed approach is shown in Figure 3. The model possesses 109,878,533 trainable parameters.

The model is compiled and trained with the learning rate of 0.00001 with Adam optimizer and batch size 16 on 50 epochs. Categorical cross-entropy loss is determined during the training and validation process to evaluate the performance of the model. Other evaluation parameters used to evaluate the model’s performance are accuracy, precision, recall, and F1-score. Adam optimizes the learning of the model by adjusting the historical gradient information of the parameters [51]. This results in efficient adaption according to the specific data. Adam leads to fast convergence by keeping the gradients recorded and updating the bias value at each step. The gradient $G_{t}$ at each step *t* with the loss function $\mathrm{Loss}(\theta_{t})$ is computed using Equation (2).

(2) $$G_{t}=\nabla_{\vartheta }\mathrm{Loss}\left(\theta_{t}\right)$$

The mean of the gradients ${\mathrm{MG}}_{t}$ by Adam at each step is determined by Equation (3).

(3) $${\mathrm{MG}}_{t}=\beta_{1}{\mathrm{MG}}_{t-1}+\left(1-\beta_{1}\right)G_{t}$$

${\mathrm{MG}}_{t-1}$ represents the previous value of the mean of the gradient.
$G_{t}$, as computed in Equation (2), is the gradient value at time step *t*.
$\beta_{1}$ is the decay rate during the computation of the mean of the gradients.

The uncentered variance ${\mathrm{VC}}_{t}$ of the gradients is computed by Equation (4).

(4) $${\mathrm{VC}}_{t}=\beta_{2}{\mathrm{VC}}_{t-1}+\left(1-\beta_{2}\right)G_{t}^{2}$$

${\mathrm{VC}}_{t-1}$ is the previous value of the uncentered variance.
$G_{t}^{2}$ represents the element-wise square value of the gradient at each step.
$\beta_{2}$ is the decay value during the computation for the uncentered variance.

Bias correction is performed during training to set the values of ${\mathrm{MG}}_{t}$ and ${\mathrm{VC}}_{t}$ as they are inclined towards 0 during the initialization of training. Equation (5) and Equation (6) represent the bias correction ${\mathrm{MG}}_{t}^{\prime }$ and ${\mathrm{VC}}_{t}^{\prime }$ for ${\mathrm{MG}}_{t}$ and ${\mathrm{VC}}_{t}$, respectively.

(5) $${\mathrm{MG}}_{t}^{\prime }=\frac{{\mathrm{MG}}_{t}}{1-\beta_{1}^{t}}$$

(6) $${\mathrm{VC}}_{t}^{\prime }=\frac{{\mathrm{VC}}_{t}}{1-\beta_{2}^{t}}$$

when dealing with a multiclass classification problem, the loss is computed using categorical cross-entropy loss. This loss function computes the difference between the probability distribution of predicted labels (obtained using SoftMax) and true encoded labels. Categorical cross-entropy loss for the single instance is computed using Equation (7).

(7) $$\mathrm{Loss}\left(y,y^{\prime }\right)=-\sum_{n}y_{n}\log\left(y_{n}^{\prime }\right)$$

The value of $y_{n}$ is 1 if the true class is n and zero otherwise, according to the encoded labels. $y_{n}^{\prime }$ is the predicted probability of the class by the model.

Evaluation parameters accuracy, recall, precision, and F1-score are computed to elaborate the performance of the model. Equations (8)–(11) illustrate the computation of these evaluation parameters.

(8) $$\mathrm{Accuracy}=\frac{\mathrm{TP}+\mathrm{TN}}{\mathrm{TP}+\mathrm{TN}+\mathrm{FP}+\mathrm{FN}}\times 100$$

(9) $$\mathrm{Recall}=\frac{\mathrm{FP}}{\mathrm{FP}+\mathrm{TN}}\times 100$$

(10) $$\mathrm{Precision}=\frac{\mathrm{TP}}{\mathrm{FP}+\mathrm{TN}}\times 100$$

(11) $$F1-\mathrm{Score}=\frac{2\times \mathrm{Precision}\times \mathrm{Recall}}{\mathrm{Precision}+\mathrm{Recall}}\times 100$$

TP, TN, FP, and FN represent the true positive, true negative, false positive, and false negative values, respectively.

## 4. Results and Discussion

The model is evaluated and assessed on the samples of two publicly available Android malware datasets.

### 4.1. Dataset Acquisition

The first dataset is CIC-AndMal2017 [52]. This dataset contains samples collected from Android apps with different kinds of malware. The data are classified into four malware classes and a collection of benign data as well. For each malware class, the samples include the malware of different families that come under the category. Table 2 describes the composition of malware classes in the dataset.

**Figure 4:**
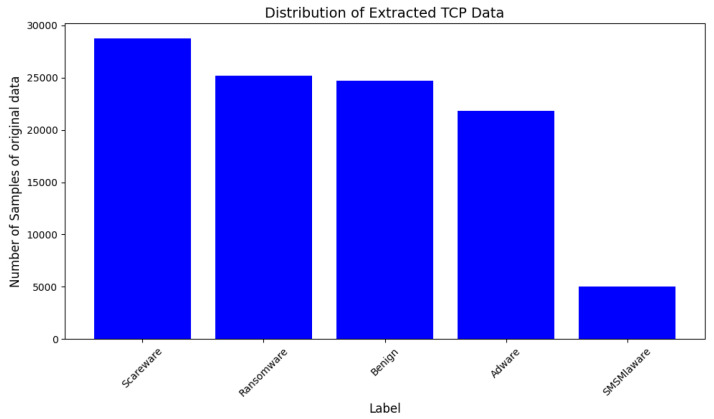
Distribution of original TCP traffic data samples of Dataset-1.

**Figure 5:**
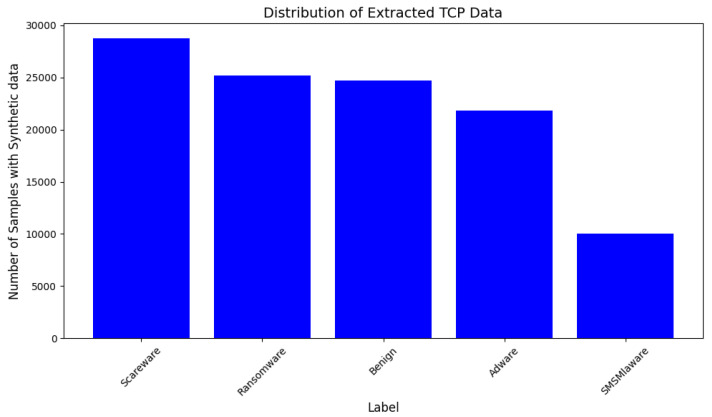
Distribution of synthetic TCP traffic data samples of Dataset-1.

A customized dataset with TCP protocol as per the problem statement is extracted from the network traffic dataset. This dataset is a comprehensive dataset of the network traffic capturing the state of the system at three stages: first, the dataset is captured instantly after installation of the malware; second, 15 min before restarting the Android device; and, lastly, 15 min after the device restarts. The data are compiled in the form of packet capture (PCAP) files. The customized dataset is a composite of samples from each class keeping a lesser number of samples from the SMS Malware class for generating the synthetic data and analysis of mutant malware by the model. The dataset is extracted for the TCP protocol. The distribution of samples of each class in the original data is represented in Figure 4 and those with synthetic samples are shown in Figure 5.

The second dataset is CIC-AAGM2017 [53]. These data are similar to that of the first dataset and capture the network traffic data. This dataset is composed of three classes. Table 3 illustrates the composition of this dataset.

**Figure 6:**
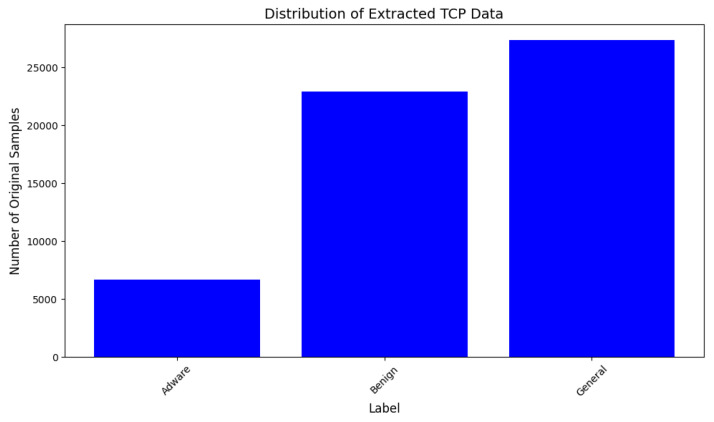
Distribution of original TCP traffic data samples of Dataset-2.

**Figure 7:**
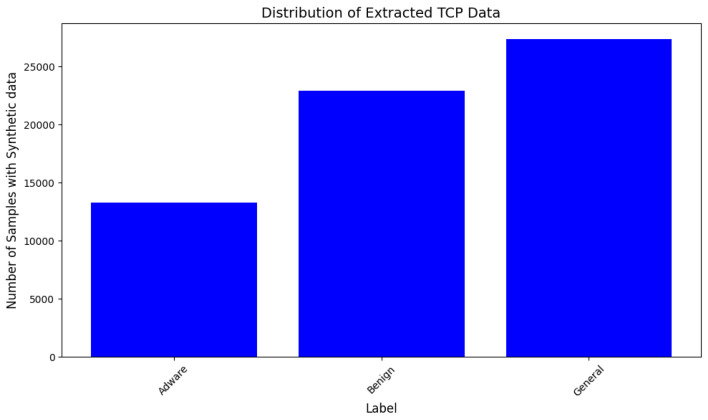
Distribution of synthetic TCP traffic data samples of Dataset-2.

This dataset is also a comprehensive dataset of PCAP (Packet Capture) files. The dataset is customized to be used for the Syn-detect model. TCP protocol packets are extracted and samples of the Adware class are less represented in the customized dataset, which makes it eligible for synthetic sample generation and augmentation. The distribution of samples of each class in the original data is represented in Figure 6 and those with synthetic samples are shown in Figure 7.

### 4.2. Deployment and Results

**Figure 8:**
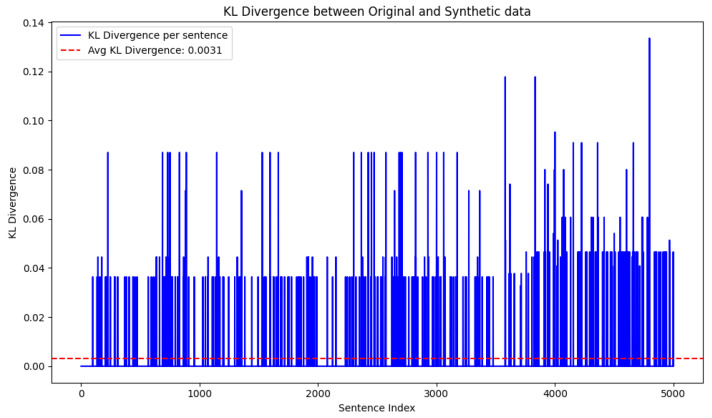
KL divergence between original and synthetic TCP traffic data samples.

The quality of the synthetic data generated by GPT-2 is validated by the KL divergence. The results of the KL divergence between the original and synthetic samples of Dataset-1 are shown in Figure 8. An average KL divergence value of 0.003 indicates that the synthetic data generated by GPT-2 are closely similar to the original data. Still, variations can be observed in the plot, which demonstrates the diversity in synthetic data. Overall, KL divergence reflects that the synthetic samples are likely realistic and diverse in comparison to the actual traffic data.

KL divergence can help to identify potential biases in synthetic data generation by comparing the probability distributions of the original traffic data and the synthetic traffic data. A high KL divergence implies that the synthetic data do not closely reflect the characteristics of the original data, which could indicate that the synthetic data generation model is biased or does not capture important facets of the real data [54]. On the other hand, a very low KL divergence, as 0.003 here, suggests that the synthetic data are very similar to the original data, implying less bias in terms of distribution. The synthetic data creation option for traffic data is low since the samples do not reflect meaningful English language. So, GPT-2 in this regard is suitable for identifying and understanding the patterns and generating diversified synthetic data [55].

**Figure 9:**
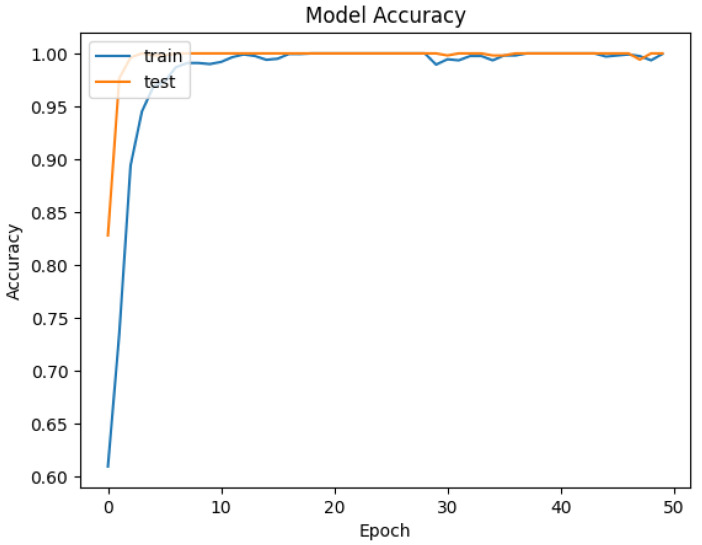
Trend of accuracy of the Syn-detect model for Dataset-1.

**Figure 10:**
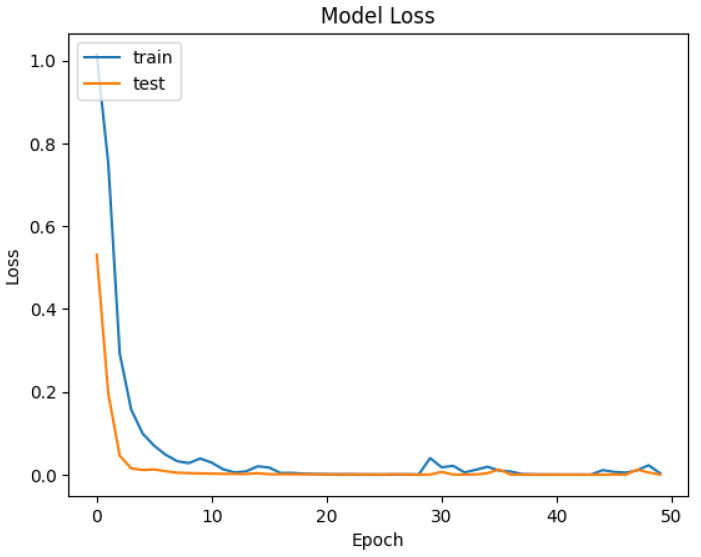
Trend of loss of the Syn-detect model for Dataset-1.

**Figure 11:**
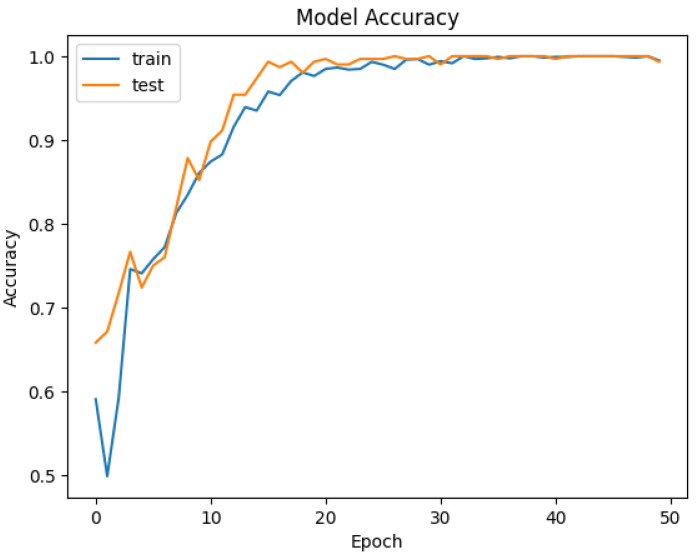
Trend of accuracy of the Syn-detect model for Dataset-2.

**Figure 12:**
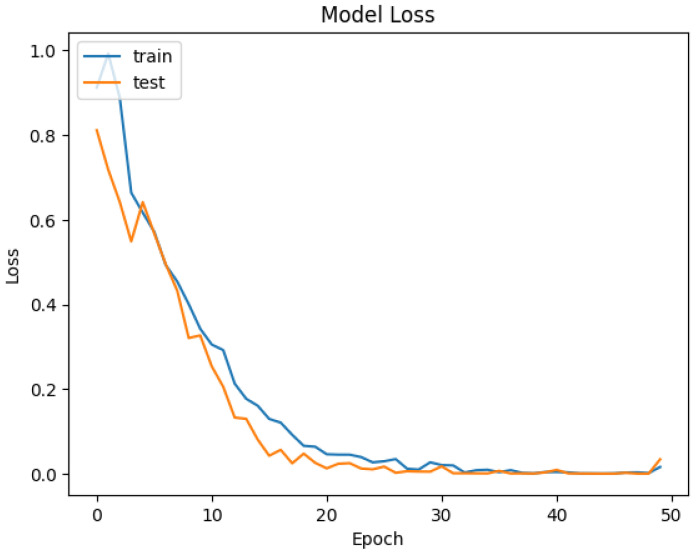
Trend of loss of the Syn-detect model for Dataset-2.

The developed datasets are preprocessed and fed into the model for tokenization. The tokenizer does its job of generating the input IDs and attention mask to be fed into the adapted BERT model for classification. The distribution of data for training and testing is kept at 80/20. Of the data, 80% is used for training of model, and 20% is used for validation. An 80/20 split was chosen to ensure a sufficient amount of data for training while reserving enough validation data to evaluate the model’s performance. Synthetic data were included in both the training and test sets. This was to evaluate how well the model generalizes to both real and synthetically generated attack patterns. Including synthetic data in the test set allowed us to assess the model’s ability to generalize to new, synthetic attack patterns. The dataset is trained and validated over 50 epochs and keeping a batch size of 16. For each epoch, the precision, recall, and accuracy are calculated to see the trend of learning by the model. The results of accuracy and loss are represented in Figure 9 and Figure 10 for Dataset-1 (CICAndMal2017) and in Figure 11 and Figure 12 for Dataset-2 (CIC-AAGM2017), respectively. Accuracy and loss for both datasets follow the trend across 50 epochs representing the increase in accuracy and decline in loss, illustrating the good learning of the model for classifying the malware.

In Figure 9, the training accuracy of the model for the CICAndMal2017 dataset is represented as blue and validation accuracy is represented as red. The accuracy of models improves as the model training starts, from 83% to 99%; then, it becomes consistent around the 15th epoch. The testing accuracy follows the same trend of increase in accuracy being consistent with the training curve at the 15th epoch. Figure 10 represents the loss curve decline as the model trains and then validates and converges to a very low value of loss.

Table 4 demonstrates the progress of the model step by step across 50 epochs towards convergence for Dataset-1.

In Figure 11, the same pattern is observed for the achievement of accuracy during training and validation of the model with CICAndMal2017. An increase in accuracy is observed until the 33rd epoch; then, it becomes consistent, achieving 98% throughout the 50 epochs. The loss declines with training and validation until it is reduced to a minute value following the 50 epochs, as depicted in Figure 12.

Table 5 demonstrates the progress of the model step by step across 50 epochs towards convergence for Dataset-2.

**Figure 13:**
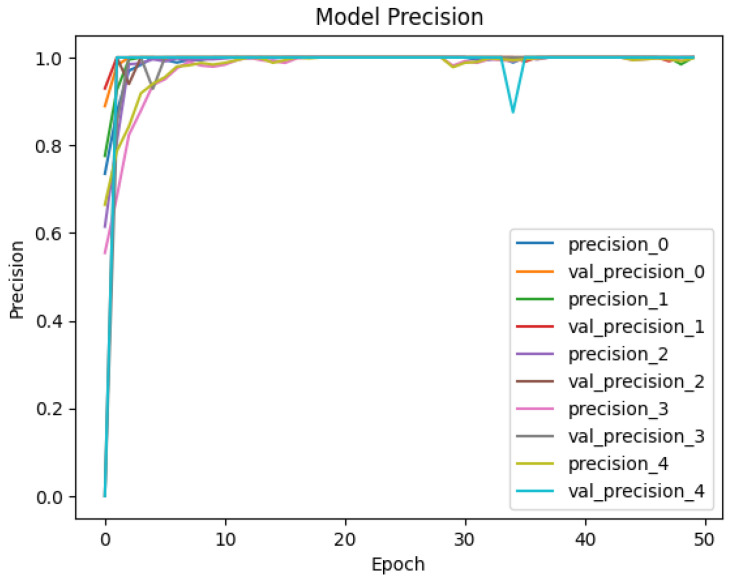
Trend of achieved precision of the Syn-detect model for Dataset-1.

**Figure 14:**
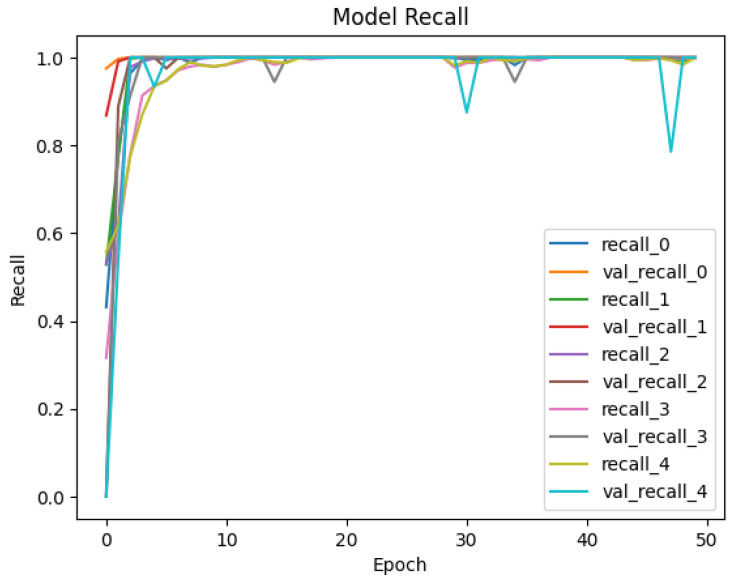
Trend of achieved recall of the Syn-detect model for Dataset-1.

**Figure 15:**
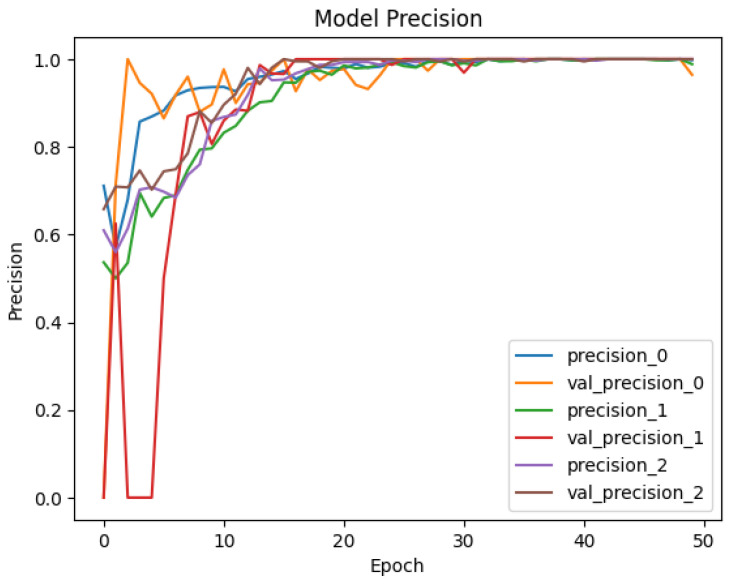
Trend of achieved precision of the Syn-detect model for Dataset-2.

**Figure 16:**
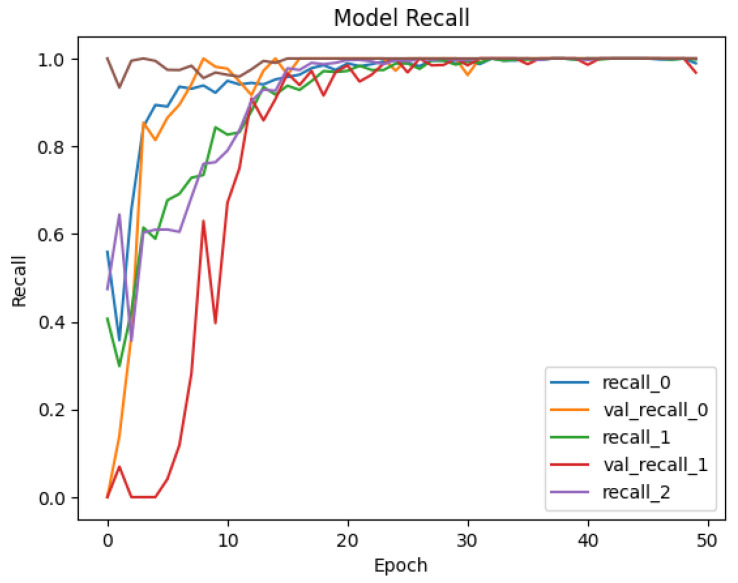
Trend of achieved recall of the Syn-detect model for Dataset-2.

To see the learning pattern throughout the model training and validation, the precision and recall of each class are monitored across 50 epochs as well. Figure 13 and Figure 14 illustrate the precision and recall trends for Dataset-1, and Figure 15 and Figure 16 illustrate the same for the second dataset. The precision values monitored during the training and validation of the model for Dataset-1 (CICAndMal2017) are represented in Figure 13. Each class is represented with its assigned Label ID 0-4. The precision value of each class during training and validation illustrates the inclination toward 1, illustrating the learning of the model as epochs progress toward 50. Figure 14 illustrates the recall values of each class as the model trains and validates. The pattern illustrates that the model can classify maximum classes of malware as it approaches the 50th epoch. Figure 15 represents the precision trend of the model with Dataset-2 (CIC-AAGM2017). The progress of the model represents that the model converges at the 30th epoch; then, a consistent curve illustrates the ability of the model to identify most of the malware classes until the 50th epoch.

The individual precision, recall, and F1-score of each class in Dataset-1 are represented in Table 6. These values represent the average values of precision, recall, and F1-score achieved across the 50 epochs. The weighted values of precision, recall, and F1-score achieved by Dataset-1 show the overall achieved values. The results are represented in Table 6.

The individual precision, recall, and F1-score of each class in Dataset-2 is represented in Table 7. These values represent the average values of precision, recall, and F1-score achieved across the 50 epochs. The weighted values of precision, recall, and F1-score achieved by Dataset-2 show the overall achieved values. The results achieved by Dataset-2 are represented in Table 7.

**Figure 17:**
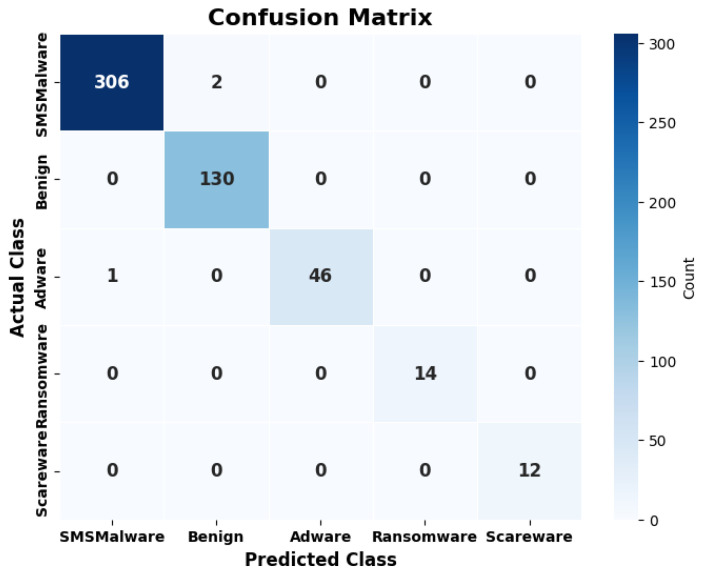
Confusion matrix of predictions for Dataset-1.

**Figure 18:**
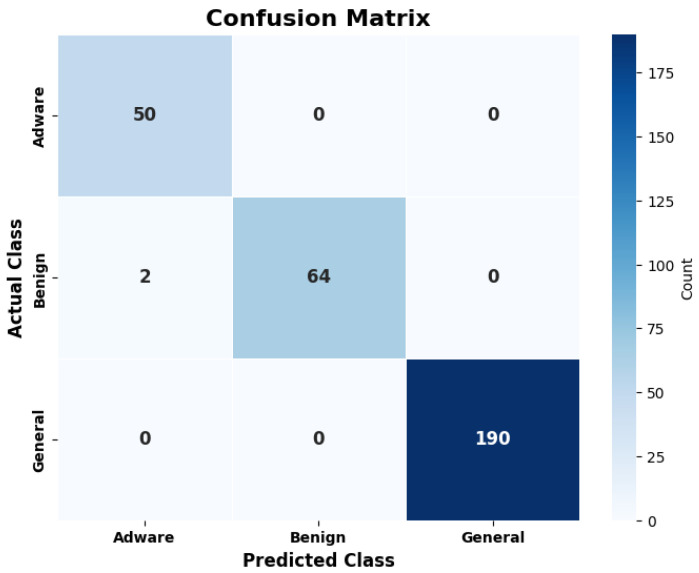
Confusion matrix of predictions for Dataset-2.

The model is evaluated on a test dataset for the predictions. The test data contain samples of original and synthetic TCP traffic data. Two sets of test data from Dataset-1 and Dataset-2 are created. Figure 17 illustrates the results of Dataset-1, and Figure 18 shows the results of Dataset-2 in the form of a confusion matrix.

On the prediction dataset, the model shows an accuracy of 99.8% for Dataset-1 and 99.3% accuracy for Dataset-2. The MCC values achieved over predictions are 0.99 for Dataset-1 and 0.98 for Dataset-2. The MCC value near 1 shows the good predictions made by the model. So, the Syn-detect model demonstrates substantial performance. In the context of malware detection and classification, false positives and false negatives can have significant impacts, even if the model achieves high accuracy. A false positive may result in unnecessary blocking that may lead to the user’s loss of trust in the system. A false negative may result in a substantial security breach. A relatively small number of false positives could cause noteworthy trouble for users in real-time systems. Similarly, false negatives would cause more malware to be misclassified as benign. This may result in more malware going undetected, putting systems at risk. Since the accuracies of 99.8% and 99.3% are significantly high in the Syn-detect model, the users are at lesser risk.

## 5. Comparison of Results and Discussion

To elaborate on the performance of the model, an ablation study was conducted by executing the model without synthetic traffic data. The model attained an accuracy of 98.2%. Results of the study are shown in Table 8 for Dataset-1 and Table 9 for Dataset-2. The findings support the effectiveness of the proposed model, as a decline in performance was observed with lower precision, recall, and F1-score values when synthetic data were not used.

With Dataset-2, the model attained an accuracy of 97.8%. The attained precision, recall, and F1-score also declined, as illustrated in Table 9.

The Syn-detect model is compared with the previous studies that deal with the imbalanced dataset for the classification of the malware. Different techniques have been used for data balancing or extraction of the nominal features so that the impact of imbalanced data on the performance of the model can be reduced. Table 10 provides a comparison of the Syn-detect model with the previous studies, and the results signify the contribution made by the model to improve the classification of the malware.

Table 10 presents a comprehensive comparison with recent studies, highlighting the implication of the Syn-detect model. Each of these studies employs diverse methods and datasets for Android malware classification. The results of each study are summarized in the table, which validates that Syn-detect outperforms all other methods. This accentuates the innovative contribution of the current study to the field of cybersecurity.

## 6. Conclusions and Future Work

Android, being the most popular operating system, is prone to cyberattacks. Detection and classification of malware has been the focus area of research throughout the process. The detection classification methods started with signature-based methods, progressed to machine learning-based methods, and finally to AI-based methods. However, this research has always been intuitive because of the dynamic nature of malware, which makes it difficult for the models to identify the mutant malware to a great extent. In this research, we targeted the network traffic data with a synthetic sample generation strategy so that the model can be trained on the synthetic samples as well. The Syn-detect model generates the synthetic samples with GPT-2 for TCP protocol-based samples and classifies the malware with an adapted BERT model. The model’s performance is assessed through extensive experimentation on two datasets. CIC-AndMAL2017 presented a weighted accuracy of 99.6% across 50 epochs and CIC-AAGM2017 showed a weighted accuracy of 95%. On the prediction dataset, the model showed an accuracy of 99.8% accuracy for CIC-AndMAL2017 and 99.3% accuracy for CIC-AAGM2017. The MCC values achieved over predictions are 0.99 for CIC-AndMAL2017 and 0.98 for CIC-AAGM2017. These impressive results illustrate that the Syn-detect model is a substantial addition to malware detection and classification research. In the future, we are aiming to develop more algorithms for malware detection and classification by unpacking the code structure and dynamic analysis. The generalizability of the model to other types of malware or network traffic can be explored in the future, which may help to evaluate the adaptability and flexibility of the model. Also, this research opens avenues for creating a secure environment for IoT-based systems through other means, including edge computing and federated learning.

## Figures and Tables

**Table 1:** Attributes of the network traffic.

Attribute	Significance
Source IP	The IP address of the sender device, which originates the traffic
Destination IP	The destination of the traffic data represents the IP address of the receiver.
Source Port	The port number used by the Sender for transmitting the data
Destination Port	The port number designated by the receiver to receive the data
Protocol	The communication protocol used for the communication between the sender and receiver

**Table 2:** Composition of Dataset-1.

Malware Class	Samples Collected	Impact of Malware
Adware	104	Adware displays advertisements on the Android device
Ransomware	101	Malicious programs that stop access to system resources
Scareware	102	Scareware generates fake security alerts for users
SMS Malware	109	Malware that spreads through SMS to mobile phones

**Table 3:** Composition of Dataset-2.

Malware Class	Apps	Impact on the Android Device
Adware	20	It attacks the security of the system by displaying malicious advertisements to the users
General Malware	150	Composed of different families of malware such as FakeAV, FakeFlash, and Penetho
Benign	1500	Non-malicious Apps

**Table 4:** Model progress on epoch steps for Dataset-1.

Epoch	Training Loss	Training Accuracy	Validation Loss	Validation Accuracy
0	1.015	0.610	0.531	0.828
1	0.753	0.735	0.196	0.977
2	0.293	0.894	0.046	0.996
3	0.157	0.945	0.015	1.000
4	0.099	0.968	0.012	0.998
5	0.070	0.973	0.013	0.998
6	0.048	0.987	0.008	1.000
7	0.033	0.991	0.005	1.000
8	0.028	0.991	0.004	1.000
9	0.039	0.990	0.003	1.000
10	0.029	0.992	0.003	1.000
11	0.013	0.996	0.002	1.000
12	0.006	0.999	0.002	1.000
13	0.008	0.997	0.002	1.000
14	0.020	0.994	0.004	1.000
15	0.017	0.995	0.001	1.000
16	0.004	0.999	0.001	1.000
17	0.004	0.999	0.001	1.000
18	0.002	1.000	0.001	1.000
19	0.002	1.000	0.001	1.000
20	0.001	1.000	0.001	1.000
21	0.001	1.000	0.001	1.000
22	0.001	1.000	0.001	1.000
23	0.001	1.000	0.001	1.000
24	0.001	1.000	0.000	1.000
25	0.001	1.000	0.000	1.000
26	0.001	1.000	0.001	1.000
27	0.001	1.000	0.000	1.000
28	0.000	1.000	0.000	1.000
29	0.040	0.989	0.000	1.000
30	0.018	0.994	0.007	0.998
31	0.021	0.993	0.000	1.000
32	0.006	0.997	0.000	1.000
33	0.012	0.997	0.001	1.000
34	0.019	0.993	0.004	0.998
35	0.010	0.998	0.013	0.998
36	0.008	0.998	0.000	1.000
37	0.001	1.000	0.000	1.000
38	0.001	1.000	0.000	1.000
39	0.000	1.000	0.000	1.000
40	0.000	1.000	0.000	1.000
41	0.000	1.000	0.000	1.000
42	0.000	1.000	0.000	1.000
43	0.000	1.000	0.000	1.000
44	0.011	0.997	0.000	1.000
45	0.007	0.998	0.001	1.000
46	0.005	0.999	0.000	1.000
47	0.011	0.997	0.012	0.994
48	0.023	0.993	0.005	1.000
49	0.003	0.999	0.000	1.000
**Weighted**	0.057	0.980	0.018	0.996

**Table 5:** Model progress on epoch steps for Dataset-2.

Epoch	Training Loss	Training Accuracy	Validation Loss	Validation Accuracy
0	0.912	0.590	0.811	0.658
1	0.992	0.498	0.718	0.671
2	0.888	0.592	0.642	0.717
3	0.664	0.746	0.549	0.766
4	0.617	0.741	0.642	0.724
5	0.571	0.758	0.568	0.819
6	0.493	0.772	0.495	0.819
7	0.454	0.813	0.433	0.819
8	0.401	0.834	0.321	0.878
9	0.343	0.861	0.327	0.898
10	0.305	0.874	0.254	0.898
11	0.292	0.883	0.206	0.974
12	0.213	0.916	0.133	0.974
13	0.177	0.939	0.130	0.974
14	0.161	0.935	0.081	0.974
15	0.130	0.958	0.043	0.993
16	0.121	0.954	0.057	0.993
17	0.092	0.970	0.025	0.993
18	0.066	0.981	0.048	0.997
19	0.064	0.976	0.026	0.993
20	0.046	0.985	0.013	0.993
21	0.045	0.986	0.024	0.997
22	0.045	0.984	0.025	0.997
23	0.040	0.985	0.012	0.997
24	0.027	0.993	0.011	1.000
25	0.030	0.990	0.017	1.000
26	0.035	0.985	0.002	1.000
27	0.012	0.996	0.006	1.000
28	0.010	0.997	0.005	1.000
29	0.027	0.990	0.005	0.997
30	0.021	0.994	0.018	1.000
31	0.020	0.992	0.001	1.000
32	0.003	1.000	0.001	1.000
33	0.009	0.997	0.001	1.000
34	0.010	0.997	0.001	1.000
35	0.004	0.999	0.007	1.000
36	0.009	0.997	0.001	1.000
37	0.002	1.000	0.001	1.000
38	0.001	1.000	0.001	1.000
39	0.004	0.998	0.003	1.000
40	0.004	0.999	0.009	1.000
41	0.003	0.999	0.001	1.000
42	0.001	1.000	0.001	1.000
43	0.001	1.000	0.001	1.000
44	0.001	1.000	0.000	1.000
45	0.001	1.000	0.000	1.000
46	0.003	0.999	0.002	1.000
47	0.003	0.998	0.000	1.000
48	0.002	1.000	0.000	1.000
49	0.016	0.995	0.034	1.000
**Weighted**	0.168	0.929	0.134	0.950

**Table 6:** Evaluations of the Syn-detect model with Dataset-1.

	SMS Malware	Benign	Adware	Ransomware	Scareware	Weighted
**Precision**	0.9973	0.9982	0.9788	0.9756	0.9775	0.9855
**Recall**	0.9993	0.9972	0.9773	0.9722	0.9627	0.9817
**F1-Score**	0.9983	0.9977	0.9780	0.9739	0.9700	0.9836

**Table 7:** Evaluations of the Syn-detect model with Dataset-2.

	Adware	Benign	General	Weighted
**Precision**	0.9484	0.8775	0.9439	0.9233
**Recall**	0.9313	0.8028	0.9932	0.9091
**F1-Score**	0.9398	0.8385	0.9679	0.9154

**Table 8:** Evaluations of the model without synthetic samples in Dataset-1.

	SMS Malware	Benign	Adware	Ransomware	Scareware	Weighted
**Precision**	0.9397	0.9945	0.9582	0.9728	0.9767	0.9683
**Recall**	0.9365	0.9947	0.9622	0.9667	0.9873	0.9694
**F1-Score**	0.9381	0.9946	0.9602	0.9697	0.9819	0.9689

**Table 9:** Evaluations of the model without synthetic samples in Dataset-2.

	Adware	Benign	General	Weighted
**Precision**	0.8488	0.9418	0.9814	0.9240
**Recall**	0.8338	0.9247	0.9973	0.9186
**F1-Score**	0.8413	0.9332	0.9893	0.9212

**Table 10:** Comparison with recent studies.

Reference	Model	Result	Dataset Used
[56]	FAMCF model based on the few-shot learning-based classification of malware to deal with insufficient labeled samples in the dataset based on the features related to static analysis.	Ensemble accuracy—Unobfuscated: 95.44%; Obfuscated: 94.39%	CICInvesAndMal2019 dataset
[57]	The LightGBM (Light Gradient Boosting Machine) model performs feature extraction to identify the significant features for the classification of Android Malware Samples.	Accuracy: 98.64% to 98.71%; Reduction in Training Time: 80 to 28 s.	CCCS-CIC-AndMal-2020
[23]	DCM-GIFT model is based on feature selection on gray-scale images of Malware signatures identified from traffic data, solving the problem of imbalanced classification results in Android software.	Recall: 95.99%; Average Recall: 98.37%; Average F1-Measure: Adware 98.78%, Benign 96.97%, Ransomware 99.35%, Scareware 96.91%, SMS Malware 99.84%	CIC-AndMal2017 and CCCS-CIC-AndMal-2020
[30]	MalBERT, based on BERT (Bidirectional Encoder Representations from Transformers) executes a static analysis on the source code of Android applications using preprocessed features for malware classification.	Binary Accuracy: 0.9761; Multi-classification Accuracy: 0.9102	Androzoo public dataset
Syn-detect Model	A two-stepped hybrid model having the process of creation of the synthetic TCP malware traffic data having malicious content with GPT-2 and classification of the malware using a fine-tuned LLM BERT with the inclusion of classification layers.	Dataset-1: 99.5%; Accuracy Weighted Precision: 0.9855; Weighted Recall: 0.9817; Weighted F1-Score: 0.9836; Dataset-2: 95%; Accuracy Weighted Precision: 0.9233; Weighted Recall: 0.9091; Weighted F1-Score: 0.9154	CIC-AndMal2017 and CIC-AAGM2017

## Data Availability

Datasets used in this research are available publicly at https://www.unb.ca/cic/datasets/index.html (accessed on 18 April 2024).
